# Resonant soft X-ray scattering reveals cellulose microfibril spacing in plant primary cell walls

**DOI:** 10.1038/s41598-018-31024-1

**Published:** 2018-08-20

**Authors:** Dan Ye, Sarah N. Kiemle, Sintu Rongpipi, Xuan Wang, Cheng Wang, Daniel J. Cosgrove, Esther W. Gomez, Enrique D. Gomez

**Affiliations:** 1 Department of Chemical Engineering, The Pennsylvania State University, University Park, PA 16802 United States; 2 Department of Biology, The Pennsylvania State University, University Park, PA 16802 United States; 3 Advanced Light Source, Lawrence Berkeley National Laboratory, 1 Cyclotron Road, Berkeley, CA 94720 United States; 4 Department of Biomedical Engineering, The Pennsylvania State University, University Park, PA 16802 United States; 5 Department of Materials Science and Engineering and Materials Research Institute, The Pennsylvania State University, University Park, PA 16802 United States

## Abstract

Cellulose microfibrils are crucial for many of the remarkable mechanical properties of primary cell walls. Nevertheless, many structural features of cellulose microfibril organization in cell walls are not yet fully described. Microscopy techniques provide direct visualization of cell wall organization, and quantification of some aspects of wall microstructure is possible through image processing. Complementary to microscopy techniques, scattering yields structural information in reciprocal space over large sample areas. Using the onion epidermal wall as a model system, we introduce resonant soft X-ray scattering (RSoXS) to directly quantify the average interfibril spacing. Tuning the X-ray energy to the calcium L-edge enhances the contrast between cellulose and pectin due to the localization of calcium ions to homogalacturonan in the pectin matrix. As a consequence, RSoXS profiles reveal an average center-to-center distance between cellulose microfibrils or microfibril bundles of about 20 nm.

## Introduction

Primary cell walls in plants are constructed of stiff cellulose microfibrils that are 3 to 5 nm wide and several microns long,^1,2^ and that are embedded in a soft, hydrated matrix of pectin and hemicellulose^3–5^. Microfibrils may exhibit semi-regular packing (*i.e*., be evenly spaced), as in celery parenchyma^6^ and collenchyma^7^; or microfibrils may be more dispersed, as in onion epidermal walls^2,8^ and maize stem parenchyma^9^. Microfibril packing may change developmentally^10,11^ and may also change dynamically, as when microfibrils are passively realigned during tensile stretching of the cell wall^12,13^. The physical connections of microfibrils with adjacent microfibrils and with the matrix are considered to be important determinants of primary cell wall mechanics and the ability for growth (*i.e*., extensibility)^14–17^, yet some aspects of the complex spatial organization of cellulose microfibrils remain challenging to characterize. In particular, methods to quantify the nanoscale spacing between microfibrils in primary cell walls are limited^2,18^, hampering efforts to connect the microstructure of plant cell walls to their macroscopic mechanical properties and to cell growth.

Micrographs from atomic force microscopy (AFM)^1,2,12,19–21^, scanning electron microscopy (SEM)^2,22^, and transmission electron microscopy (TEM)^23–25^ can visualize cellulose microfibrils at the surfaces of primary cell walls. The higher moduli of cellulose microfibrils with respect to the matrix leads to imaging contrast in AFM. SEM relies on differences in electron backscattering to visualize the topology, whereas TEM relies on rotary shadowing of a replica; both EM techniques benefit from partial removal of matrix polymers to enhance microfibril visualization. The presence of calcium associated with carboxylic acids in homogalacturonan, the predominant pectic polysaccharide, provides an opportunity to generate contrast based on differences in elemental composition using analytical TEM techniques. Previous work has demonstrated variation of calcium in plant cell walls using electron energy-loss spectroscopy (EELS),^26,27^ although the resolution was limited such that microfibrils are not apparent.

In principle, software such as SOAX^28^ and Ridge Detection^29^ can identify fibrils in acquired images and enable quantitative estimates of microfibril organization. Spacing between microfibrils can be extracted from peaks in pair distribution functions or from dominant spatial frequencies in Fourier transformed images. Despite the clear impact of direct imaging of cell wall structure, these imaging techniques typically yield information over areas of 1 μm^2^ to 100 μm^2^, thereby limiting the statistical significance of quantitative results even with multiple images. Indeed, AFM and SEM imaging of cell walls revealed an average 3 to 5 nm fibril diameter^2,6,30,31^, but there are no reports on the interfibril spacing.

A relatively new technique, sum-frequency generation (SFG) spectroscopy, is sensitive to the lateral packing of cellulose microfibrils when the packing distance is within the SFG coherence length (several hundred nanometers) over large areas (ca. 30,000 μm^2^)^32^. The ratio of the CH_2_ peak to the OH peak in SFG spectra is proportional to packing distances of cellulose microfibrils^32,33^. Nevertheless, other factors can also affect this ratio and it is not yet feasible to extract values for the spacing from SFG spectra.

Small angle X-ray scattering (SAXS) and small angle neutron scattering (SANS) are well suited to reveal mesoscale periodicities within various materials. As an approach complementary to microscopy, scattering reveals density correlations in reciprocal space from “bulk” (e.g. 1 mm thick, 40,000 μm^2^ area) samples. SAXS has been used to extract cellulose microfibril orientation in terms of spiral angle from wood cell walls^34–36^, epidermal cell walls of *Avena* coleoptiles^37^, and primary cell walls of *Chara* and *Arabidopsis*^38^. When cell walls exhibit a high degree of order, scattering can also provide the spacing between microfibrils, but primary walls rarely have sufficient order. Diameter and lateral spacing of cellulose microfibrils in spruce wood (a secondary cell wall) have been determined using SANS^39^, where a spacing was found to be about the size of individual microfibrils (3 to 4 nm). Thus, this spacing is obtained from microfibrils that are packed in bundles. X-ray scattering and SANS experiments have also shown that an average interfibril spacing can be extracted from well-ordered celery collenchyma wall^40,41^ and woody cell walls of dicots^42^; again a spacing from dry samples is extracted as 3 to 4 nm. In primary cell walls of *Arabidopsis* inflorescence stems, SANS experiments rely on contrast between D_2_O and cell wall components to reveal a spacing of approximately 4 nm that corresponds to microfibrils tightly packed in bundles.^18^ Much of the scattering profile is dominated by a power law with an exponent between 2.5 and 4, which most likely represents the complex structure of matrix polymers. Revealing spatial correlations between matrix polymers or between poorly organized microfibrils or microfibril bundles remains a challenge, in particular beyond the spacing of tightly packed microfibrils.

A recently developed technique, resonant soft X-ray scattering (RSoXS), relies on tuning the X-ray energy to enhance contrast. For soft materials, tuning the X-ray energy between 200 eV and 2000 eV (in contrast to hard X-ray scattering that typically relies on 8–12 keV X-rays) can access the absorption edges of constituent elements of biological materials such as carbon, nitrogen, and oxygen. By modulating incident X-ray energies to match absorption edges of different chemical motifs, scattering contrast can be enhanced. In addition, the short mean free paths of soft X-rays make RSoXS an ideal technique to characterize thin samples (hundreds nm to microns). Over the last decade, RSoXS has been transformative to elucidate the microstructure of polymer thin films through enhanced scattering contrast based on chemistry differences^43–47^. Recently, RSoXS has been applied to study the internal structure of casein micelles through examination of calcium ions^48,49^. Nevertheless, the application of RSoXS to plant cell walls has not been demonstrated.

Here, we examine the packing of cellulose microfibrils of onion epidermal cell walls using RSoXS. Onion epidermis is an experimental platform to link mechanical properties to microfibril spatial organization due to the ease of accessing large areas of primary cell wall^12,50–52^. Scattering data at the carbon (ca. 285 eV) and calcium (ca. 349 eV) edges from onion yields information regarding the cell wall internal structure. Scattering profiles from the carbon edge appear to be dominated by the structure of the cuticle, whereas scattering at the Ca edge is dominated by contrast between cellulose microfibrils and Ca bound to the pectin matrix. We find that treatment with Ca enhances contrast, while degradation of the pectin matrix with pectate lyase decreases Ca content and reduces contrast. Thus, we attribute features from scattering profiles at the Ca edge to an approximately 20 nm spacing within the cell wall. Although the resolution of RSoXS at the Ca edge is limited by the circa 3.6 nm X-ray wavelength, we demonstrate that RSoXS can quantify the center-to-center spacing between isolated cellulose microfibrils or between microfibril bundles in the polysaccharide matrix of plant cell walls.

## Results and Discussion

**Figure 1:**
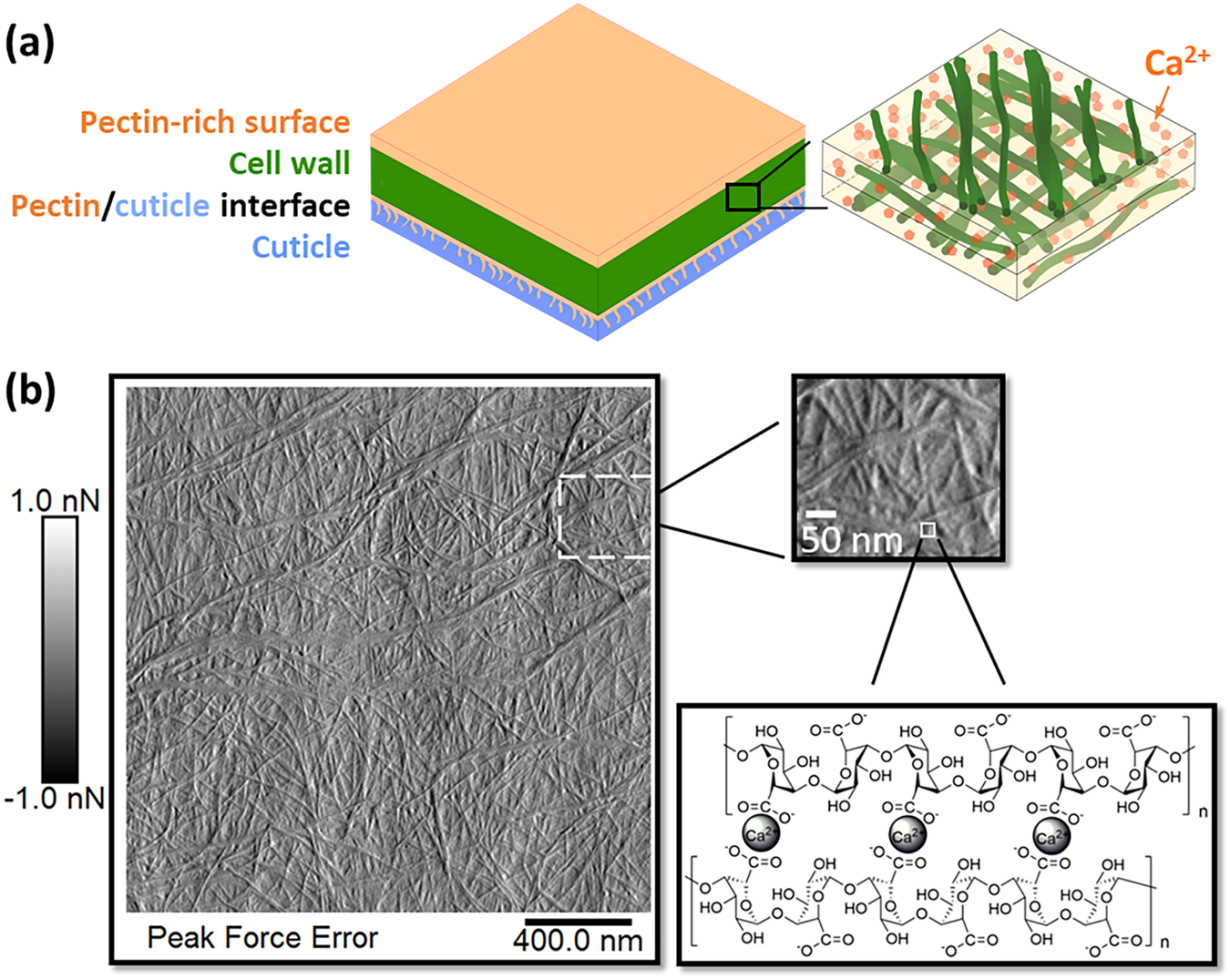
(**a**) Schematic of different layers within onion peels. Cellulose microfibrils are dispersed within the cell wall as single microfibrils and aggregates in a pectin-rich matrix. (**b**) AFM image of 11^th^ scale onion epidermis peel (dried, in air) and schematic of calcium crosslinking of homogalacturonan, the major component of the pectin matrix surrounding the cellulose microfibrils. AFM resolution of microfibrils in dried cell walls is much poorer than in hydrated walls, but because the RSoXS measurements required dry samples, we show an AFM image of a dried wall. The 11^th^ scale (counting inwards from the outermost fleshy scale) was selected for this study because it is thinner than older (outer) scales.

Onion abaxial epidermal cell walls are easy to peel to expose the cell wall surface. A cuticle layer lies underneath, as highlighted in schematics of onion epidermis shown in Fig. 1a and in Figs S1 and S2 of the Supplementary Information. Because of the different moduli of cellulose and pectin components of the cell wall, cellulose microfibrils can be visualized through AFM images of the peak force error, as shown in Fig. 1b. Celluloses, in the form of long microfibrils, are anisotropically aligned in the wall, and some microfibrils aggregate into bundles. In between fibrils, the matrix is mostly composed of homogalacturonan^21^. When de-methylesterified, homogalacturonan can be crosslinked through calcium ions. Overall, AFM images reveal microfibrils that are packed in a semi-regular fashion that are spaced by tens of nanometers, as depicted in Fig. 1a.

**Figure 2:**
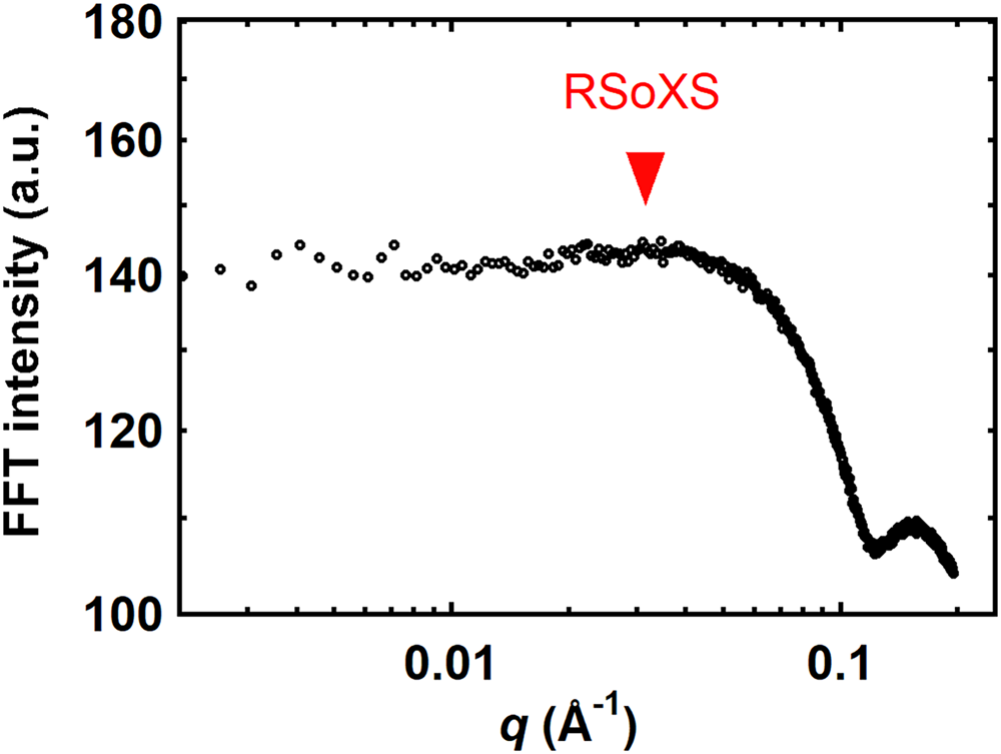
Fast Fourier transform (FFT) intensity as a function of *q* calculated from the AFM image shown in Fig. 1 of unextracted 11^th^ scale onion epidermal peel. The arrow denotes the average interfibril spacing determined from RSoXS.

Quantitative analysis of spatial features in AFM micrographs is possible through image analysis toolkits. SOAX^28^ and ImageJ^53^ were used sequentially to identify microfibrils and produce binary images containing foreground microfibrils and background surrounding matrix. Fast Fourier Transforms (FFTs) were subsequently performed on binary images to obtain quantitative spatial information. Integrating reciprocal space intensities over all azimuthal angles yields profiles that reveal multiple features, as shown in Fig. 2. A shoulder or broad peak is apparent near *q* = 0.04 Å^−1^ and another peak is visible near *q* = 0.15 Å^−1^. These features correspond to length scales of about 16 nm and 4 nm, respectively (*d* = 2π/*q*). As shown in Fig. S3 of the Supplementary Information, the high *q* peak is consistent with the form factor of a long rod with a diameter of 6.6 nm. The 6.6 nm diameter observed in the dried cell wall is larger than previously reported values for single microfibrils of 3.5 nm determined from real-space analyses of AFM micrographs of hydrated onion walls^2^. We attribute this difference to extensive bundling of microfibrils^2^, collapse of matrix onto microfibril surfaces upon drying, and broadening by the AFM tip^21^. Alternatively, the high *q* peak in the FFT can be attributed to the center-to-center distance of microfibrils within bundles, with microfibrils that are approximately 4 nm in diameter. The low *q* peak cannot be explained by the form factor of microfibrils, and we therefore assign this peak to the interfibril distance (*i.e*. correlation peak of the structure peak). Although we can estimate interfibril spacings using this approach, image processing can sometimes miss some of the cellulose microfibrils and limited sampling areas (*e.g*. 2 μm × 2 μm) limit statistical confidence of extracted values.

In contrast to microscopy techniques, scattering yields structural information averaged over a large sample area; the beam size of an X-ray source is 10^4^ to 10^5^ μm^2^. As shown in Fig. S4 of the Supplementary Information, we examined the microstructure of onion abaxial primary cell wall using SAXS. Scattering data is similar to background scattering (Fig. S4a of the Supplementary Information), and subtracted data show a *q*^−4^ dependence (Fig. S4b of the Supplementary Information). Thus, scattering in the hard X-ray regime is mostly dominated by voids, surface roughness, or large structures; no structural information at the nanoscale can be extracted from onion epidermis. The lack of internal structure information from SAXS could be due to multiple factors. Maximizing scattering signal requires a sample thickness near the inverse of the attenuation coefficient. As shown in Fig. S5a of the Supplementary Information, the ideal sample thickness for scattering experiments at 10 keV is around 2 mm for cell walls. Onion epidermal walls from the 11^th^ scale have a thickness of about 1 μm, or 2000 times lower than the ideal thickness. Furthermore, the contrast between cellulose and pectin is small, because the densities of cellulose (1.599 g/cm^3^)^54^ and pectin (1.543 g/cm^3^)^55^ are similar. Thus, contrast is limited at 10 keV.

Alternatively to SAXS, RSoXS is an ideal tool to characterize the morphology of thin films with chemical specificity. As shown in Fig. S5a of the Supplementary Information, the attenuation length of cell wall polymers near the carbon K-edge (~285 eV) is about 1 μm, which is comparable to the thickness of onion epidermis. Furthermore, by tuning the incident energy to match absorption edges associated with different chemical moieties, scattering contrast can be enhanced from differences in the X-ray absorption spectra. Consequently, structural features that are not apparent at energies away from absorption edges (off-resonance) could be apparent at resonance. For onion cell walls, we can enhance scattering contrast between pectin matrix and cellulose microfibrils due to the calcium ions in the pectin matrix.

**Figure 3:**
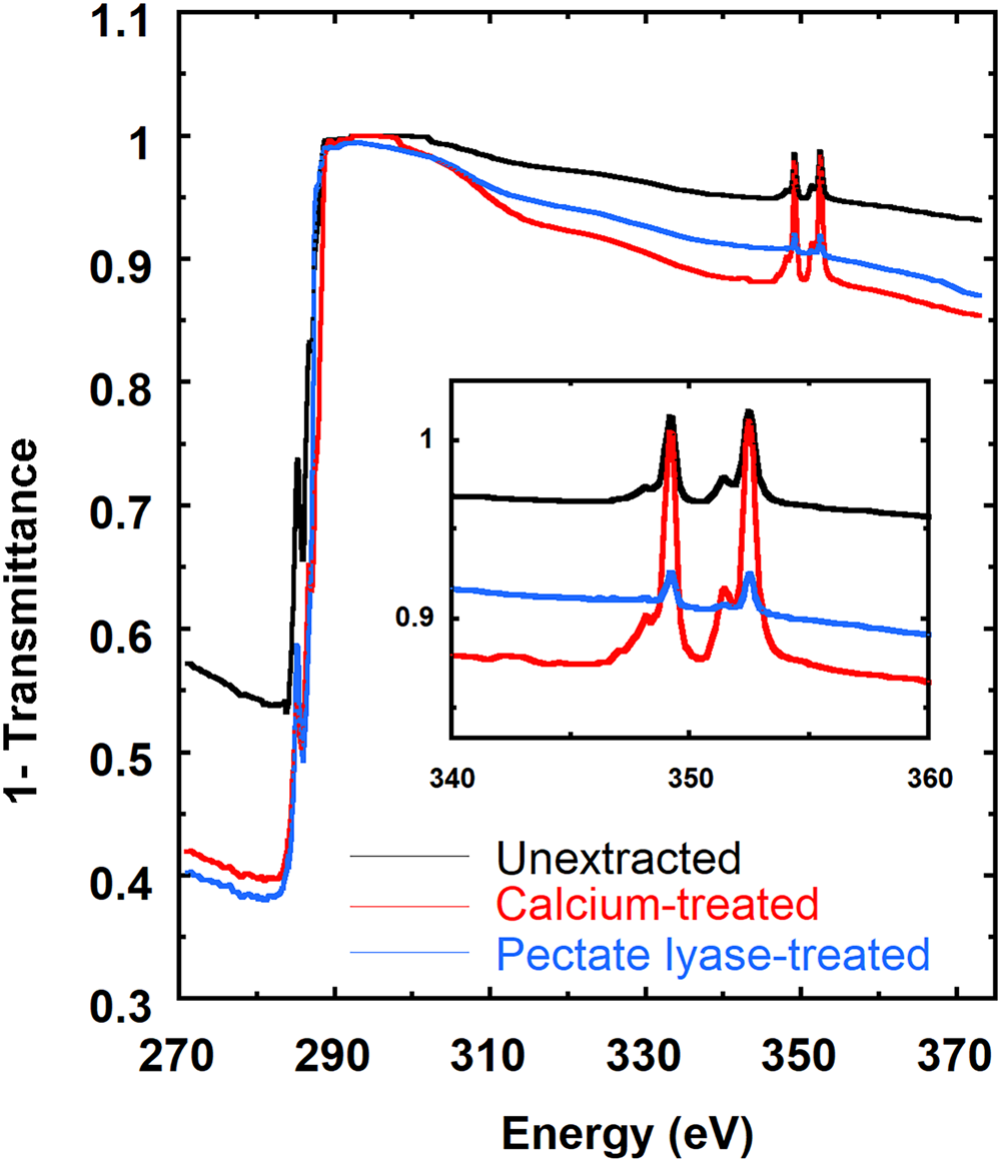
NEXAFS spectra of unextracted, calcium-treated, and pectate lyase-treated onion 11^th^ scale epidermis. Inset: NEXAFS spectra between 340 eV and 360 eV.

Differences in the X-ray absorption spectra can be examined using Near Edge X-ray Absorption Fine Structure (NEXAFS) experiments. As shown in Fig. 3, spectra were collected for unextracted, calcium-treated, and pectate lyase-treated onion epidermis samples from the carbon K-edge (270–320 eV) to the calcium L-edge (330–360 eV). Near the carbon K-edge, the peak at 285 eV corresponds to the 1 s(C=C) → ${{\pi }^{\ast }}_{C=C}$ transition. For unextracted and calcium-treated samples, this feature corresponds to the C=C bond in the epicuticular wax layer or residual wall proteins. Because pectate lyase cleaves pectin within the cell wall, the C=C bond signature from the pectate-lyase treated sample could also arise from enzymatic degradation products due to *β*-elimination^56^. At energies above 288 eV, X-ray absorption increases significantly due to the C step edge, making the sample transmittance nearly zero for the thickness of the onion 11^th^ scale (1.1 µm) until ca. 300 eV where the X-ray absorption drops from the increase in the attenuation length (Fig. S5a). As a consequence, interpretation of NEXAFS spectra is challenging between 288 eV and 300 eV.

Near the calcium L-edge, the peaks at 349.3 eV (L_2_) and 352.6 eV (L_3_) are due to the 2p → 3d transitions of calcium ions^48,57^. Figure 3 shows that Ca is clearly present in native (unextracted) cell walls. To enhance scattering contrast, we add Ca ions to the pectin network through treatment with 2 mM CaCl_2_, which results in higher X-ray absorption at 349.3 eV and 352.6 eV. Furthermore, we can remove Ca ions by cleaving homogalacturonan using pectate lyase, and thereby rinse away Ca along with homogalacturonan fragments. Treatment with pectate lyase effectively digests homogalacturan although it does not remove all pectins; quantification of uronic acids and monosaccharide analyses reveals that pectate lyase releases 190 μg sugar per mg of starting material (145 μg galacturonic acid), while the control (untreated) releases 27 μg per mg of starting material (3 μg galacturonic acid). As a consequence, pectate lyase-treated samples show less intense Ca peaks in the NEXAFS spectra. Varying the Ca content in our samples should affect scattering contrast and therefore RSoXS profiles.

A key advantage of RSoXS is the ability to modulate the contrast by tuning the X-ray energy. We can predict scattering contrast (Δ*n*Δn^*^/λ^4^) between two components, such as between cellulose and calcium-treated pectin, by calculating differences in the refractive indices, $n(\lambda )=1-\delta (\lambda )-i\beta (\lambda )$, of the components as given by^43,47,58,59^:

1 $$\frac{{\rm{\Delta }}n(\lambda ){\rm{\Delta }}{n}^{\ast }(\lambda )}{{\lambda }^{4}}=\frac{{(\delta {(\lambda )}_{i}-\delta {(\lambda )}_{j})}^{2}+{(\beta {(\lambda )}_{i}-\beta {(\lambda )}_{j})}^{2}}{{\lambda }^{4}}$$

where *i* and *j* denote two species, and *β* and *δ* are the absorptive and dispersive component of the refractive indices, respectively. The dispersive component *δ* is calculated from *β* through the Kramers-Kronig relations^58,60,61^. Although *β* can be predicted from the chemical composition using KKcalc,^62^ we merge experimentally obtained NEXAFS spectra near the C K-edge and Ca L-edge with KKcalc predictions to obtain accurate estimates of *β* from 10 eV to 30 keV. Methods for obtaining NEXAFS spectra of the constituent materials, here cellulose and pectin, are described below^43,58,59,63^.

**Figure 4:**
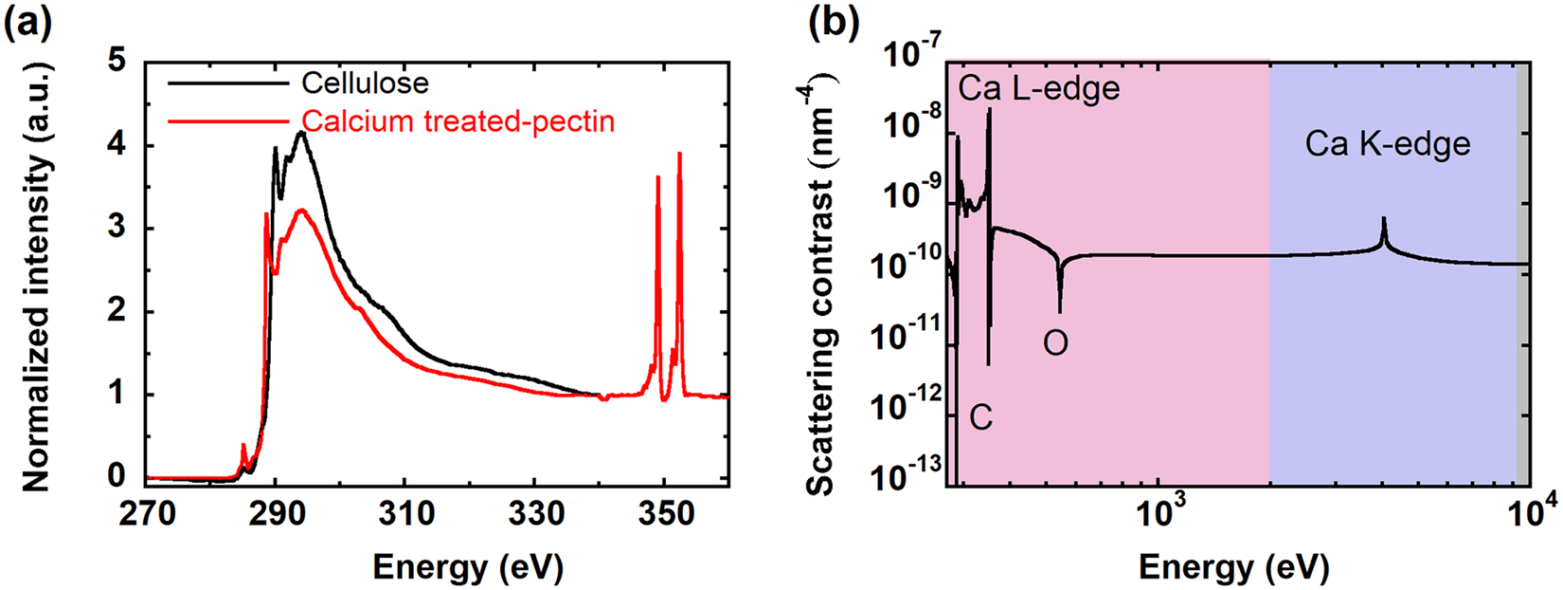
(**a**) Experimentally measured NEXAFS spectra of cellulose (previously reported^64^) and calcium-treated pectin. (**b**) Scattering contrast between cellulose and calcium treated-pectin as a function of energy for soft (pink), tender (purple), and hard (grey) X-rays.

We use published Partial Electron Yield (PEY) NEXAFS data^64^ to obtain *β* and *δ* for cellulose (Fig. 4a). For pectin, we use citrus pectin that is approximately 60% methylesterified. Although the NEXAFS spectra near the C K-edge is likely similar for both onion and citrus pectin, the apparent Ca content of citrus pectin is small, likely due to a high esterification level^65^. We thus combine the C K-edge NEXAFS spectra from citrus pectin with the total electron yield (TEY) spectra from calcium-treated 11^th^ scale onion at the Ca L-edge (Fig. S6a of the Supplementary Information). To account for cellulose and hemicellulose in the epidermal cell walls, the merged TEY spectrum was then scaled near the calcium L-edge by assuming pectin makes up 40% of the cell wall by volume (Fig. S6b of the Supplementary Information)^66^. Combined with KKcalc, this approach yields our best estimate of the X-ray absorption spectra for calcium-treated onion pectin for a wide energy range (10 eV to 30 keV), and can consequently yield *β* and *δ*.

Using our estimates of *β* and *δ* for cellulose and pectin, we calculate the scattering contrast as a function of X-ray energy using equation 1 (Fig. 4b). In the soft X-ray regime (200 eV-2000 eV), scattering contrast between cellulose and pectin is maximized near the calcium L-edge, and is two orders of magnitude higher than the scattering contrast in the hard X-ray regime (ca. 10 keV). We therefore expect that scattering at the Ca L-edge will be dominated by the structural features associated with the dispersion of cellulose microfibrils in the pectin matrix.

**Figure 5:**
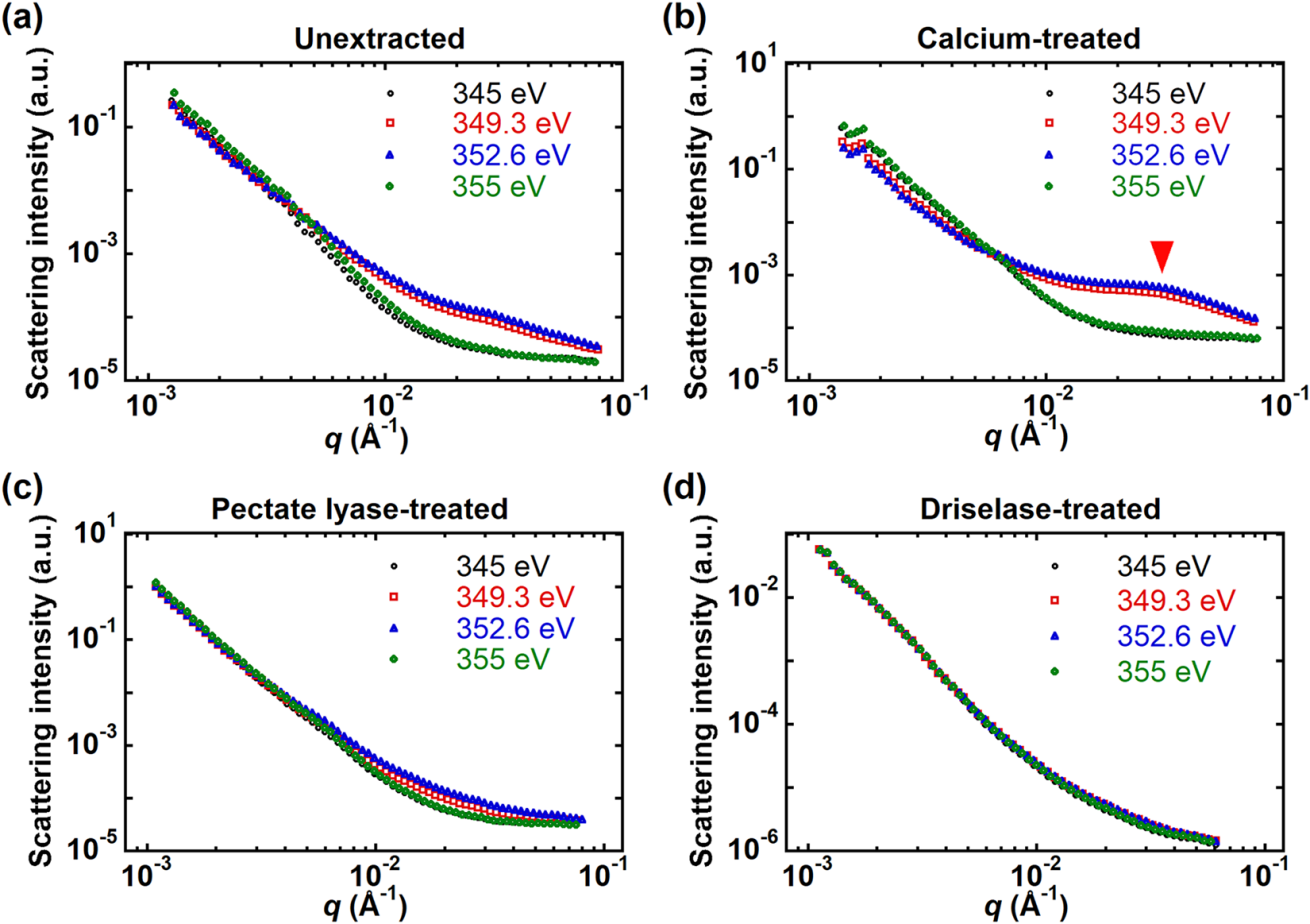
RSoXS profiles at resonance (349.3 eV and 352.6 eV) and off resonance (345 eV and 355 eV) near the calcium L-edge of (**a**) unextracted, (**b**) calcium-treated, (**c**) pectate lyase-treated, and (**d**) Driselase-treated onion 11^th^ scale epidermis. Data acquired in the transmission geometry (X-rays normal to the epidermis).

Figure S7 of the Supplementary Information shows 2D scattering images of unextracted, calcium-treated, and pectate lyase-treated epidermis samples. A slight anisotropy in the scattering is apparent, likely due to a preferred orientation of fibrils^38,67^. Figure 5 shows RSoXS profiles carried out at four energies near the calcium L-edge, two of which are on-resonance (349.3 eV and 352.6 eV) and two are off-resonance (345 eV and 355 eV). At all energies, a roughly *q*^*−4*^ power law dependence is apparent at low *q*, suggesting scattering dominated by interfaces due to significant roughness, large scale structures, or voids within the sample. In the unextracted cell walls (Fig. 5a), no other features were observed at 345 eV (pre-edge) and 355 eV (post-edge), while at resonance (349.3 eV and 352.6 eV), there is a weak peak near *q* = 0.03 Å^−1^ corresponding to a length scale of 20 nm (*q* = 2π/*d*). This length scale from RSoXS is roughly consistent with the spacing apparent from AFM (Fig. 1). Comparing RSoXS with the FFT of the AFM image, the spacing obtained from RSoXS is consistent with the broad shoulder shown in Fig. 2. The peak at higher *q* that is apparent in Fig. 2 and in SANS profiles^18^ corresponding to the 4 nm spacing of microfibrils within bundles is outside the *q* range of RSoXS data in Fig. 5 given the approximately 3.6 nm X-ray wavelength near the Ca edge. A scattering angle beyond 70 degrees would be required to observe a maximum in scattering corresponding to this spacing; currently, about 60 degrees is the practical maximum. On the other hand, the scattering peak near *q = *0.03 Å^−1^ observed at X-ray energies of 349.3 eV and 352.6 eV becomes more apparent following incubation of the sample in a solution of 2 mM CaCl_2_ (arrow, Fig. 5b), likely due to saturation of Ca^2+^ cross-linking of the unesterified homogalacturonan component of the pectin matrix. Previous AFM imaging experiments on the 5^th^ scale onion epidermal cell wall have demonstrated that addition of 10 mM CaCl_2_, followed by addition of the chelator EDTA, has little effect on microfibril properties^2^; thus, we do not expect significant changes to the structure of cell wall components following this treatment at the lower calcium concentration used in our experiments. Instead, Ca treatment enhances X-ray contrast between pectin and cellulose, thereby highlighting the microstructure composed of cellulose microfibrils dispersed in the pectin matrix. After treatment with pectate lyase, the scattering feature near *q* = 0.03 Å^−1^ disappears (Fig. 5c), as expected from the reduced Ca content and reduced scattering contrast.

We also consider the contributions to scattering results from the cuticle layer underneath the epidermal cell wall. Cuticle is composed of a waxy layer (epicuticular wax) and a layer composed of cutin^68^. Cutin is a polymeric material that is mostly composed of C16 and C18 ω-hydroxyacids linked through esterification^68,69^. To separate the scattering from cuticle, we used Driselase, which is composed of various enzymes, to remove polysaccharides, including cellulose, from the cell wall. RSoXS profiles from onion epidermis treated with Driselase and treated with both Driselase and CaCl_2_ are shown in Figs 5d and S8b of the Supplementary Information for X-ray energies near the Ca edge. The scattering feature near *q* = 0.03 Å^−1^ that is visible in unextracted samples and in samples treated with Ca is not apparent when cell wall components are removed. Thus, the structure near *q* = 0.03 Å^−1^ is not from cuticle but corresponds to structural features within cell walls.

We can further corroborate the structural origin of scattering profiles by comparing the predicted contrast to the total scattering intensity (TSI) from RSoXS experiments in a way that is typically not possible for scattering with hard X-rays (ca. 10 keV). As illustrated in Figs 1 and S1a of the Supplementary Information, the onion epidermis has a layered structure. Each layer can contribute to the total scattering. We consider the scattering between pectin and vacuum from the top surface, pectin and cellulose within the cell wall, and pectin and cuticle at the pectin/cuticle interface. The TSI from each layer is related to scattering contrast as^70,71^

2 $$TSI(E)=V{\phi }_{i,j}{\rm{\Delta }}{n}_{i,j}(E){\rm{\Delta }}{n}_{i,j}^{\ast }(E)/{\lambda }^{4}$$

where *i* and *j* represent the two components in each onion epidermis layer (*e.g*., cellulose and pectin, Fig. S1 of the Supplementary Information), *V* is the total scattering volume of the layer, and $\,{\phi }_{i,j}$ is the product of the volume fraction between domains *i* and *j*. For simplicity, we assume that the volume fraction of the two components in each layer remains constant. We take the thickness of each layer to be proportional to the volume (*i.e*., we assume each layer has a similar density). Thus, here when TSI is scaled by the layer thickness, it is proportional to the scattering contrast (${\rm{\Delta }}{n}_{i,j}(E){\rm{\Delta }}{n}_{i,j}^{\ast }(E)/{\lambda }^{4}$) between constituent components.

**Figure 6:**
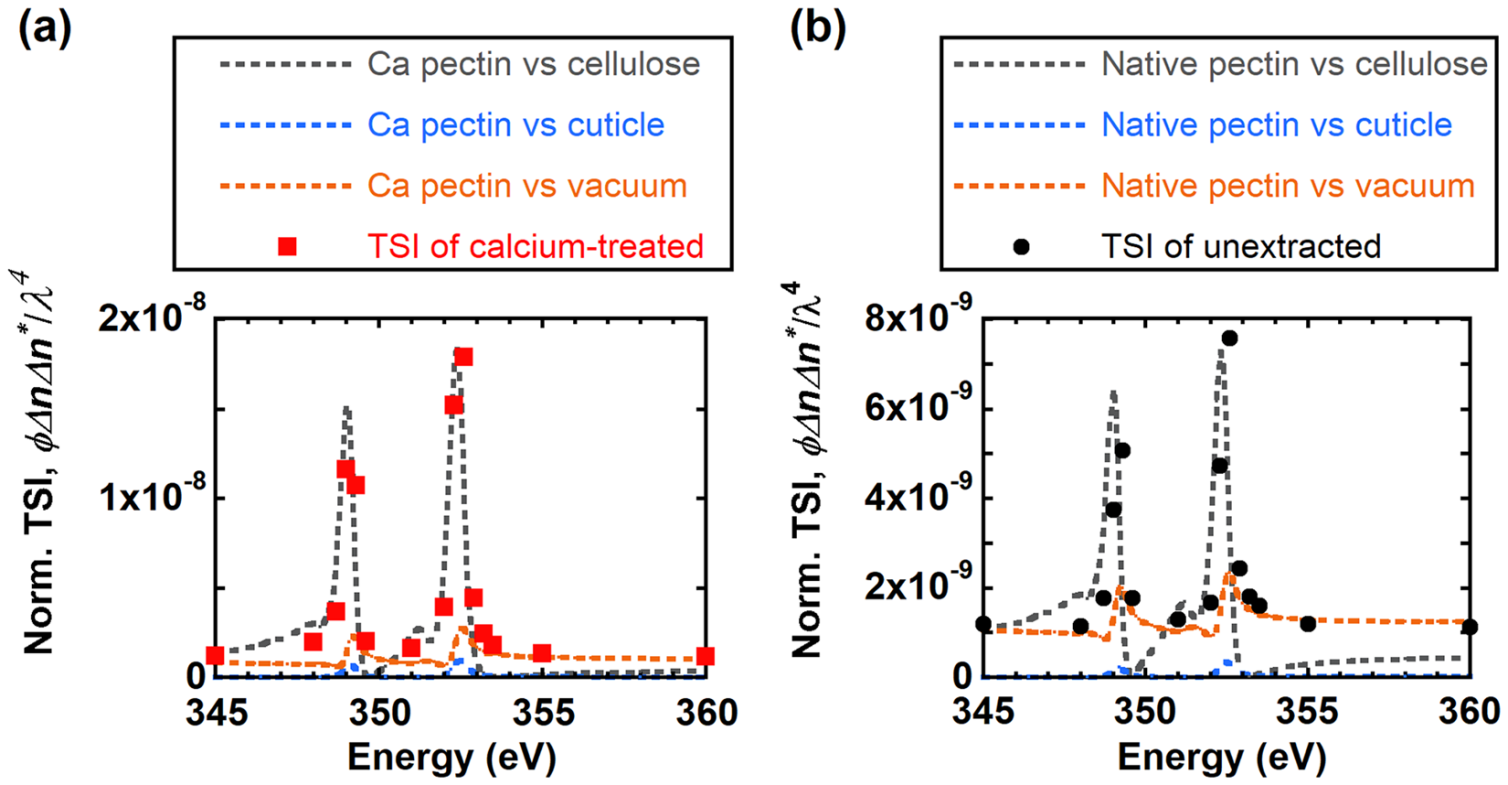
Comparison of TSI (points) of (**a**) calcium-treated and (**b**) unextracted epidermal cell wall to the product of volume fraction and scattering contrast (dotted lines) from different cell wall components near the calcium L-edge. TSI is normalized to the same value for both (**a**,**b**). Volume fraction *ϕ* is proportional to the thickness of the layer where contrast originates.

Although we lack an absolute intensity calibration to estimate values for TSI from RSoXS data, we can normalize scattering data by the direct beam flux, exposure time, and sample transmittance and then calculate TSI as ${\int }^{}I(q){q}^{2}dq$. For comparison between experimental TSIs and predicted contrast, we scale the TSI to the predicted contrast at 280 eV or 345 eV. Figure 6a shows the predicted contrast between Ca-treated pectin and cellulose, cuticle, or vacuum to the experimentally-derived TSI for the Ca-treated onion epidermis near the calcium L-edge. Details on the scattering contrast calculations are discussed in the Supplementary Information. The predicted scattering contrast between cellulose and Ca-treated pectin agrees well with the TSI from RSoXS results, and scaling TSIs cannot match the expected contrast between Ca-treated pectin and cuticle or vacuum. Furthermore, Fig. S9a of the Supplementary Information indicates that the Ca-treated sample has the highest TSI near the Ca L-edge, followed by the unextracted sample, as expected. Altogether, Figs 6a and S9a of the Supplementary Information demonstrate that the contrast between pectin and cellulose dominates scattering at X-ray energies in resonance with the Ca L-edge. The feature could either represent the form factor of cellulose microfibrils (or microfibril bundles) or a peak from the structure factor that corresponds to interfibril spacing (or spacing between microfibril bundles). Fig. S10 of the Supplementary Information shows that RSoXS scattering results do not match various cylindrical form factors given the breadth of the peak. Given that SANS shows that the spacing between microfibrils within bundles is 4 nm,^18^ we attribute the feature at *q* = 0.03 Å^−1^ shown in Fig. 5b to the average center-to-center spacing between isolated microfibrils or between microfibril bundles in cell walls, where the spacing is given by *d* = 2π/*q* and is 20 nm. This model of microfibrils dispersed in a pectin-rich matrix is consistent with AFM images and electron micrographs,^2,6,72–75^ and with cellulose-pectin contacts detected with solid state NMR in primary cell walls^4^.

We can also estimate the Ca ion concentration in the pectin matrix of the unextracted onion epidermis. Assuming the calcium-treated epidermis is saturated with Ca ions and that pectin makes up 40% of the dry cell wall, we scale the NEXAFS spectra of calcium-treated pectin near the Ca edge by 0.4 while holding the C edge spectra constant to match TSIs to the predicted contrast from cellulose and pectin in unextracted onion cell walls (Fig. 6b). This is in reasonable agreement with the decrease in the Ca NEXAFS intensity, where the ratio Ca (352.6 eV)/C (288 eV) decreases from 1.2 for Ca-treated epidermis to 0.31 for unextracted samples. Thus, we estimate that Ca ions crosslink 30% to 40% of carboxyl groups in polygalacturonic acids for unextracted epidermis from the NEXAFS spectra and by comparing the expected contrast to RSoXS intensities.

**Figure 7:**
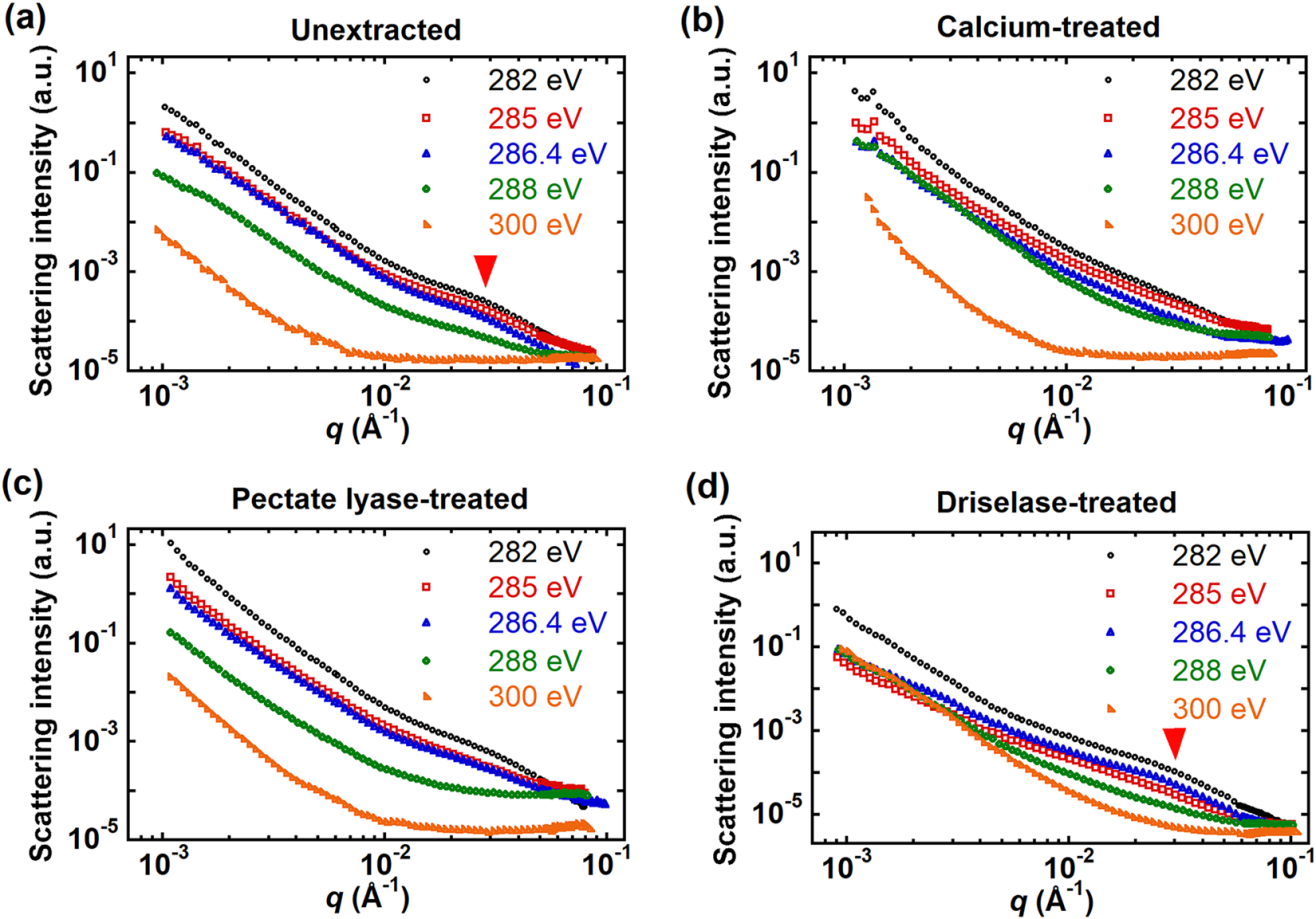
RSoXS profiles of (**a**) unextracted, (**b**) calcium-treated, (**c**) pectate lyase-treated, and (**d**) Driselase-treated onion 11^th^ scale epidermis near the carbon K-edge. Scattering profiles are shifted vertically for clarity. Data acquired in the transmission geometry (X-rays normal to the epidermis).

In addition to the Ca L-edge, we can acquire data at the C K-edge to examine the structure of plant cell walls. RSoXS data was acquired at five different energies, including one in the pre-edge region (282 eV), and four at π^*^ (285 eV, 286.4 eV and 288 eV) and σ^*^ (300 eV) resonances. Figure 7a shows a weak peak or shoulder near *q* = 0.03 Å^−1^ in the unextracted cell walls (highlighted with an arrow). After pectate lyase treatment, the scattering profile looks about the same as from unextracted tissues (Fig. 7c). The calcium treatment makes this feature less apparent (arrow, Fig. 7b). But, the feature near *q* = 0.03 Å^−1^ is still apparent after Driselase treatment (Fig. 7d), despite the removal of most cellulose and pectin. Thus, the peak near *q* = 0.03 Å^−1^ must be at least in part due to a structural feature within the cuticle layer.

**Figure 8:**
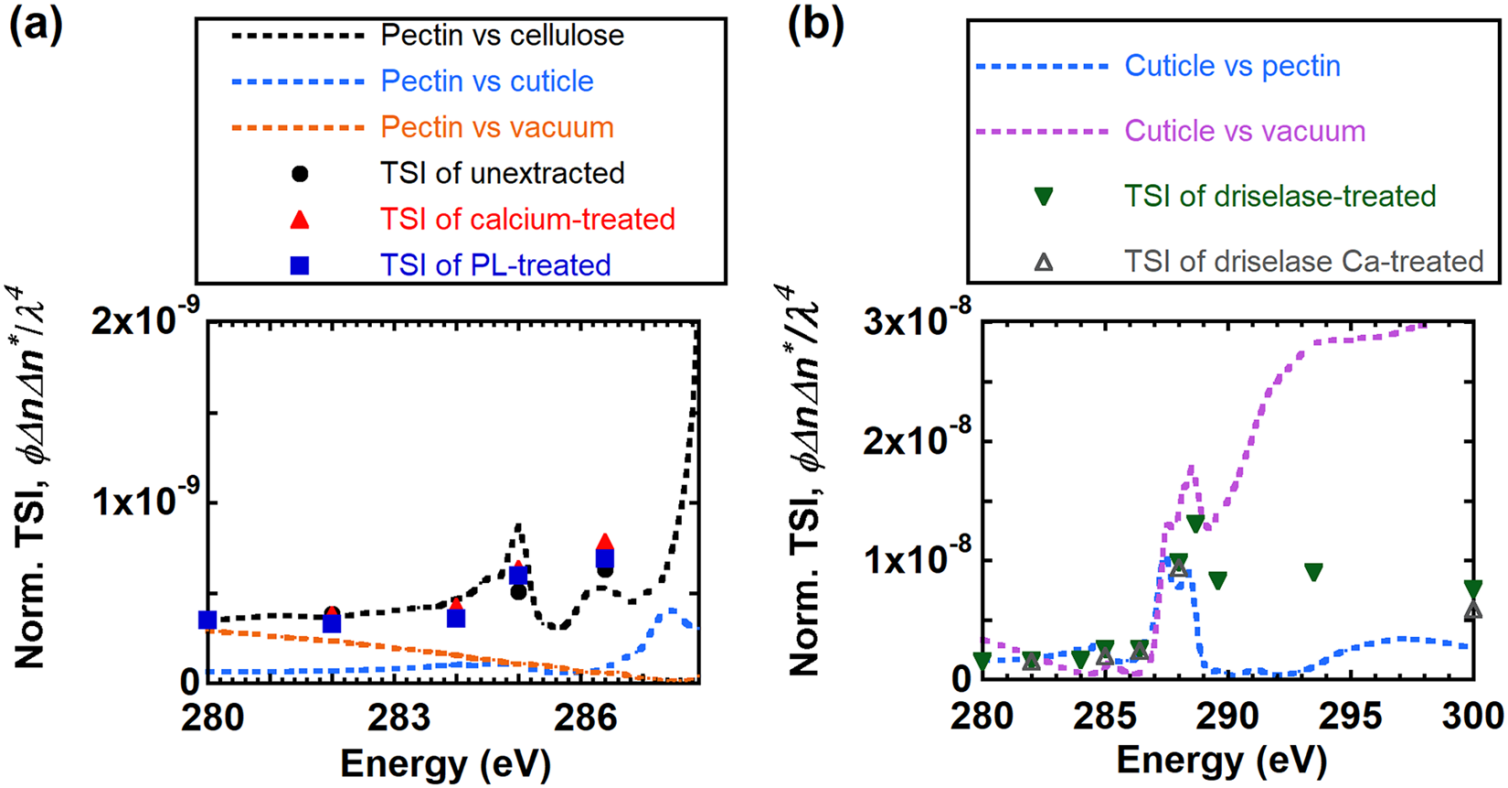
Comparison of TSIs (points) from (**a**) unextracted, calcium-treated, and pectate lyase-treated and (**b**) Driselase-treated and Driselase calcium-treated cell walls to the product of volume fraction and scattering contrast (dotted lines) from different cell wall components near the carbon K-edge. Volume fraction *ϕ* is proportional to the thickness of the layer where contrast originates.

We compare TSIs and predictions of the scattering contrast to ascertain the dominant contributions to RSoXS intensities near the carbon K-edge. Scattering contrast is calculated from the refractive indices; refractive indices of cellulose and pectin were calculated from NEXAFS spectra shown in Fig. 4 as described above. We take the NEXAFS data (Fig. S11 of the Supplementary Information) from onion epidermis treated with Driselase to calculate the refractive index for cuticle. Figure 8a compares TSIs from unextracted, pectate lyase-treated, and calcium-treated epidermis to predicted scattering contrast between pectin and cellulose, pectin and cuticle, or pectin and vacuum. These samples absorb X-rays strongly near the carbon K-edge between 288 eV and 300 eV (sample transmittance <1%), such that TSI values in this range are not accurate. Nevertheless, below 288 eV, experimental TSIs are most consistent with predicted scattering contrast between pectin and cellulose. As a consequence, for unextracted and pectate lyase-treated samples, the feature near *q* = 0.03 Å^−1^ could be from the spacing between cellulose microfibrils; although, Fig. 7d suggests a significant contribution from the cuticle layer as discussed above. Indeed, scaling the TSI could allow for a reasonable match to the contrast between pectin and cuticle. Figure 8a also shows that the contrast between pectin and vacuum (i.e., surface roughness) cannot describe the experimental scattering intensities. Thus, although multiple factors may contribute to RSoXS intensities near the C K-edge, we predict that the contrast between pectin and cellulose is highest and thus dominates scattering. For calcium-treated epidermis, the presence of calcium ions might change the density of the pectin matrix in a subtle way that reduces the contrast between pectin and cellulose such that no feature is apparent in RSoXS scattering profiles near the carbon K-edge.

For Driselase-treated samples, we consider scattering that could originate from two possibilities: a mixture of pectin and cuticle or rough cuticle with voids (Fig. S1b,c of the Supplementary Information). Although we expect that Driselase can remove all pectin, we consider a mixture of cuticle and pectin to include the possibility that Driselase cannot access some pectin impregnated into cuticle^68,76^. We thus calculate scattering between cuticle and pectin and between cuticle and vacuum and compare to experimental TSIs. Figure 8b shows that both the predicted scattering contrast between cuticle and vacuum and cuticle and pectin agree well with the TSI trend of Driselase-treated and Driselase calcium-treated samples below 288 eV. Above 288 eV, TSIs are lower than the predicted contrast between cuticle and vacuum but higher than the predicted contrast between cuticle and pectin. We therefore attribute scattering for Driselase-treated samples to originate from either voids in cuticle, where the cuticle itself has perhaps lost some density due to Driselase treatment, or from a mixture of voids in cuticle and some pectin embedded within cuticle even after Driselase treatment. Furthermore, the feature near *q = *0.03 Å^−1^ is apparent up to 286.4 eV but disappears above 288 eV, and the curvature of scattering profiles differs below and above 288 eV. These results suggest that the scattering is associated with different features below and above 288 eV, which is consistent with multiple structural features contributing to the scattering. Thus, we hypothesize that for Driselase-treated samples the feature near *q* = 0.03 Å^−1^ below 288 eV corresponds to phase separation between pectin and cuticle, but above 288 eV contrast between cuticle and voids or cuticle surface roughness dominates scattering. Evidence for intercalation of pectin and cuticle is apparent as a reticulate zone that is observed between cuticle and cell walls and the persistence of staining with ruthenium red after pectinase and cellulase treatment^77,78^. Alternatively, structure within the pectin-rich matrix, due perhaps to heterogeneity in composition of pectin or hemicellulose, could also contribute to RSoXS profiles near the C K-edge.

## Conclusions

RSoXS is a powerful tool to examine the microstructure of plant cell walls. We demonstrate that RSoXS overcomes challenges with examining thin cell walls with modest scattering contrast found in conventional SAXS experiments. Tuning the X-ray energy, and as a consequence X-ray contrast, allows various features to be highlighted in scattering profiles. At the Ca L-edge, RSoXS profiles show a broad peak corresponding to a center-to-center spacing of 20 nm between isolated cellulose microfibrils or microfibril bundles. Furthermore, comparing total scattering intensities to predicted scattering contrast from different components for various X-ray energies facilitates assignment of the structural origin of scattering profiles. This addresses a fundamental problem of X-ray scattering, the lack of uniqueness in structures that produce the same scattering profiles, by requiring that possible structures produce both the *q* and energy dependence of soft X-ray scattering. The ability to tune contrast and examine thin films makes RSoXS an invaluable tool to examine mixtures of polysaccharides and plant cell walls.

## Materials and Methods

### Sample preparation

White onion (*Allium cepa* L. *cometa*) bulbs were obtained from local grocery stores and the periclinal epidermal wall from the abaxial surface of the 11^th^ scale was prepared^79^. All peels were first washed with 0.1% Tween-20 in 20 mM HEPES buffer (pH 6.8) for 1 h using previously reported methods,^21^ and additionally, rinsed six times with distilled deionized water (ddH_2_O) to remove the detergent and other cell debris. Peels were then allowed to air dry. Calcium-treated peels were prepared by incubating in 2 mM CaCl_2_/ 20 mM Tris buffer (pH 9.5) overnight for 16 h at 37 °C with gentle shaking at 50 rpm, to bind Ca to the pectin in the cell wall. For pectate lyase-treated samples, peels were incubated with 20 μg/mL pectate lyase (Megazyme E-PCLYAN2) in 20 mM Tris (pH 9.5) with 2 mM CaCl_2_ for 16 h at 37 °C while shaking gently at 50 rpm, to digest homogalacturonan (HG). Following pectate lyase digestion, the supernatant was put through a 0.2 µm filter to remove cell debris and monosaccharide analysis was performed as previously described^18^. Driselase solutions (nominally 10 mg/mL) were prepared by adding Driselase powder (a fungal enzyme cocktail; Sigma; Cat # D9515) to 20 mM sodium acetate buffer (pH 5.5). Solutions were mixed for 30 mins, then purified through centrifugation for 5 mins at 10 g, and the supernatant was put through a 0.2 µm filter to remove any remaining particulates. Cuticles were prepared by enzymatic digestion of onion peels in 10 mg/mL Driselase solutions for 6 days at 37 °C while shaking at 50 rpm. For calcium-treated cuticles, detergent washed peels were incubated in 10 mg/mL Driselase solutions along with 2 mM CaCl_2_ for 6 days at 37 °C while shaking at 50 rpm. After the above treatments, peels were rinsed with ddH_2_O six times and then dried in air.

To measure the thickness of the pectin layer at the surface of the wall, an onion epidermal peel from the 5^th^ scale was incubated with CaCl_2_ to allow HG-Ca binding, which results in stiffening of the pectin layer, enabling detection by AFM. The epidermal peel was first washed with 0.05% Tween-20 in 20 mM HEPES buffer (pH 7.5) for 30 min. The peel was then treated with 50 µg/mL pectin methylesterase (PME) for 15 min to create free carboxyl groups on the HG backbone for Ca binding. Following PME treatment, the peel was briefly washed with the same HEPES buffer with 100 mM NaCl to remove PME proteins. Lastly, the peel was incubated with 100 mM CaCl_2_ for 1 h prior to AFM imaging in 100 mM CaCl_2_.

For near edge X-ray fine structure (NEXAFS) spectroscopy, pectin from citrus (Sigma-P9436 with 60% esterification) was dissolved in filtered DI water at a concentration of 10 mg/mL. The pectin solution was spin coated at a speed of 1500 rpm onto a silicon wafer resulting in a 60 nm thick film. Film thickness was determined using a P16 Stylus profilometer.

### Monosaccharide analysis after Pectate Lyase digestions

The monosaccharide analysis protocol was adapted from previously reported methods^80^. In brief, 1 mg of dry cell wall powder and 500 µL of 3 N methanolic hydrochloric acid were mixed and incubated at 80 °C for 16 h, cooled on ice, and dried with filtered air. Two hundred μL of 2 M trifluoroacetic acid was added to the dried residue, mixed thoroughly, incubated at 121 °C for 2 h in a sealed tube, and cooled on ice.

### Atomic force microscopy

Cell wall surfaces were scanned using a Dimension Icon AFM (Bruker, CA, USA) with ScanAsyst and the PeakForce Quantitative Nanomechanical Property Mapping operation package as previously described^21^. All images were scanned at 512 × 512 pixels (scan size 2 µm by 2 µm) and imaged with a SCANASYST-AIR (for dried walls) or SCANASYST-FLUID + tip (for hydrated walls) with a tip radius of about 2 nm. Deflection sensitivity was characterized by indenting on a rough glass slide and the spring constant was calibrated using a thermal tune method. The system parameters were as follows: peak force setpoint (800 pN), scan rate (0.7 Hz), and peak force frequency (1 kHz). SOAX software^18^ was used to identify and extract cellulose microfibrils from the peak force error AFM images. The 2D FFT function in ImageJ^41^ was then used to obtain the 2D frequency domain images. 1D profiles of the frequency domain data were calculated by taking the azimuthal average of 2D FFT images.

To estimate the thickness of the pectin layer at the surface of onion walls, a 6^th^ order plane fit was applied to an AFM height image in order to correct for sample tilting and to remove unevenness of the wall surface. The resulting image contains height values for each pixel assuming the cell wall surface is flat. A histogram of height values from the image was used to calculate the average thickness of the pectin on the surface. Outlier data points within the histogram were removed using 0.05% of the total height population as the cutoff on each end of the distribution.

### Stylus profilometry

Sample thickness was measured by a Tencor P16 Stylus profilometer. For unextracted and Driselase-treated epidermis, air-dried peels from 3 different onions were used. Six measurements were made for each peel. Thicknesses were estimated from averages of 18 measurements from three onions.

### Near edge X-ray fine structure (NEXAFS) spectroscopy

NEXAFS spectra were collected at beamline 11.0.1.2 at the Advanced Light Source (ALS), Lawrence Berkeley National Laboratory. For transmission NEXAFS, samples were mounted on 50 nm thick Si_3_N_4_ windows with a 5 mm × 5 mm silicon frame (Norcada). The angle between the sample to incident X-ray beam was maintained at 90°. Spectra from 270 eV to 360 eV covering the carbon K-edge and calcium L-edge were collected within a single scan. The transmitted X-ray intensity was recorded using a photodiode detector. NEXAFS spectra were normalized with respect to direct beam flux and blank substrate absorption. For total electron yield (TEY) NEXAFS, samples were mounted or spun coat on a silicon substrate. The angle between the plane of samples to incident X-ray beam was kept at 55°. The TEY signal was first normalized by direct beam flux. Then the background from the silicon substrate was subtracted by fitting the pre-edge data with a linear function.

### Resonant soft X-ray scattering

Washed and dried onion peels were mounted on 50 nm thick Si_3_N_4_ windows with a 5 mm × 5 mm silicon frame (Norcada). Scattering experiments were carried out at beamline 11.0.1.2 at the ALS in the transmission geometry using a charged coupled detector.^81^ Two sample to detector distances (50 mm and 150 mm) were used to cover a *q* range of 0.0013 Å^−1^ to 0.08 Å^−1^. Dark counts, solid angle, and exposure time corrections were applied to 2D images. The 1D scattering profiles were obtained by azimuthally averaging the pixel intensities from the 2D detector images using the Nika software package.^82^ Background scattering was collected from bare Si_3_N_4_ windows. A direct beam flux scan was recorded using a photodiode detector to normalize scattering data and account for flux variations with energy. Empty cell scattering was confirmed to be negligible when compared to scattering from onion epidermal peels.

### Small angle X-ray scattering

Samples were mounted on washers for transmission SAXS experiments. SAXS measurements were conducted at beamline 7.3.3 at the ALS.^83^ The sample to detector distance was about 4 meters and a 1 M Pilatus detector was used to collect scattering data. Scattering intensity was azimuthally averaged. Scattering from air was collected for the same exposure time as samples to account for background scattering.

## Electronic supplementary material

Supplementary Information

## Data Availability

The data that support the conclusions from this study are available from the corresponding authors upon request.
