# Supplementary material for: Resonant soft X-ray scattering reveals cellulose microfibril spacing in plant primary cell walls
Source: Sci Rep. 2018 Aug 20;8:12449. doi: 10.1038/s41598-018-31024-1 (PMC6102304; doi:10.1038/s41598-018-31024-1)
Supplement: Supplementary file 1 — Supplementary Information [file 41598_2018_31024_MOESM1_ESM.pdf]

Supplementary Information for

Resonant soft X-ray scattering reveals cellulose microfibril spacing in onion cell wall

Dan Ye[1], Sarah N. Kiemle[2], Sintu Rongpipi[1], Xuan Wang[2], Cheng Wang[3], Daniel J. Cosgrove[2], Esther W. Gomez[1,4]* and Enrique D. Gomez[1,5]*

[1]Department of Chemical Engineering, The Pennsylvania State University, University Park, PA 16802, United States

[2]Department of Biology, The Pennsylvania State University, University Park, PA 16802, United States

[3]Advanced Light Source, Lawrence Berkeley National Laboratory, 1 Cyclotron Road, Berkeley, CA 94720, United States

[4]Department of Biomedical Engineering, The Pennsylvania State University, University Park, PA 16802, United States

[5]Department of Materials Science and Engineering and Materials Research Institute, The Pennsylvania State University, University Park, PA 16802, United States

Table of Contents

1. **Onion epidermis structure and scattering contrast of each layer** 2
2. **Comparing FFT of AFM image to cylindrical form factors** 4
3. **Small angle X-ray scattering** 4
4. **Effect of sample thickness on scattering intensity** 5
5. **NEXAFS and scattering contrast** 6
6. **2D RSoXS Images** 7
7. **Driselase digested calcium-treated epidermis** 8
8. **Total scattering intensity and scattering contrast** 9
9. **Comparing RSoXS data to cylindrical form factors** 10
10. **References** 10

1. Onion epidermis structure and scattering contrast of each layer

As shown in Figure S1a, the onion epidermis is composed of several layers. Based on profilometry measurements (Table S1), the unextracted epidermis is 1145 ± 131 nm thick and the Drislease-treated epidermis is 152 ± 40 nm thick. Most of the cell wall materials are removed with Drislease digestion. The top 52 nm could be a mixture of pectin and cuticle (Figure S1b), if some pectin is intertwined with cuticle such that some pectin cannot be fully removed by Driselase. Alternatively, all pectin is digested completely by drislease, and some of the cuticle layer is left with holes (Figure S1c).

For epidermal peels, AFM studies indicate that there is a thin layer of pectin sitting on top of cell walls[1]. Based on the AFM height profile (Figure S2b) of the superficial pectin layer, the height distribution of pectin can be fitted to a Gaussian distribution with 43 nm as the mean and 14 nm as the standard deviation. The thicknesses of the cuticle layer and cuticle/pectin layer remain the same as the Driselase-treated sample, which leads to a 950 nm thick cell wall layer for untreated epidermal peels.

Based on the layered structure of untreated epidermis, the scattering contrast near the calcium L-edge was calculated for each layer of the epidermis (shown in Figure S1a). In the top 43 nm pectin layer, the contrast comes from pectin and vacuum. For the 950 nm thick cell wall layer, the scattering contrast is dominated by pectin and cellulose. Scattering contrast arises from pectin and cuticle in the mixed 52 nm pectin-cuticle layer. For our two models of the Driselase-treated sample shown in Figure S1b and S1c, the scattering contrast from the top 52 nm layer could either arise from cuticle and pectin (Figure S1b) or cuticle and vacuum (Figure S1c).

Table S1. Cell wall thickness measured by stylus profilometry

	Unextracted	Driselase-treated
Thickness (nm)	1145±131	152±40

*Error is standard deviation from multiple measurements (n=18).

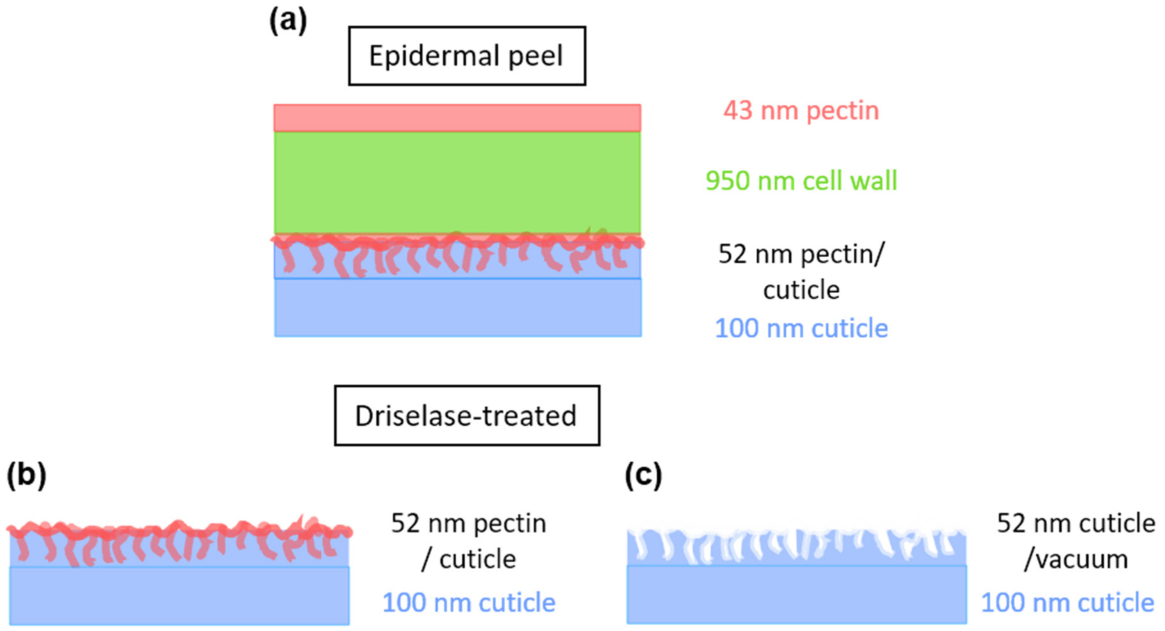

Figure S1. Schematic of different layers within (a) untreated onion epidermal peel and Driselase-treated onion epidermal peel with (b) partial removal or (c) complete removal of pectin.

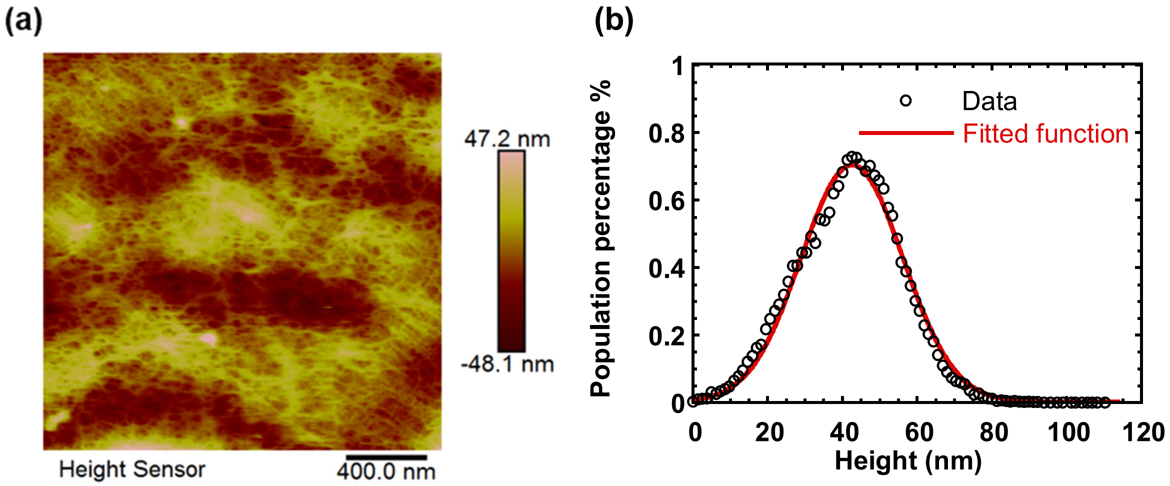

Figure S2. Top pectin layer thickness. (a) Flattened AFM height image of the superficial pectin layer of calcium and PME-treated onion 5th scale epidermis. (b) Height distribution of pectin layer and fitted Gaussian distribution with 43 nm as the mean and 14 nm as the standard deviation.

2. Comparing FFT of AFM image to cylindrical form factors

The Fourier transformed frequency spectra of the AFM image shown in Figure 1 of the main text has two features. A shoulder or broad peak is apparent near $q = 0.04$ Å^{-1}, and another peak is visible near $q = 0.15$ Å^{-1}. The feature near $q = 0.15$ Å^{-1} can be described with a cylindrical form factor with a diameter of 6.6 nm and length of 0.97 µm as shown in Figure S3.

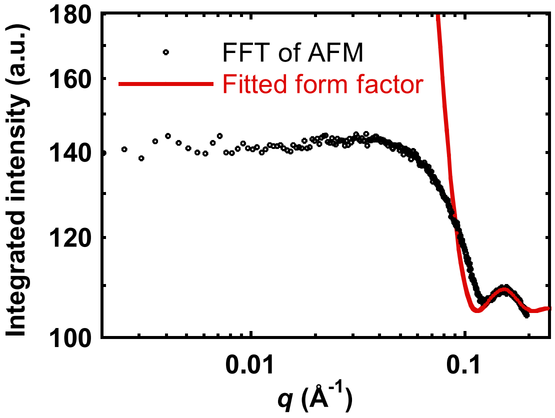

Figure S3. Fitting of the feature near $q = 0.15$ Å^{-1} in FFT intensity of the AFM image shown in Figure 1 to cylinder form factor. The fitted form factor has a diameter of 6.6 nm and a length of 0.97 µm.

3. Small angle X-ray scattering

SAXS data from unextracted epidermal peel and from the direct beam (scattering from air) are shown in Figure S4a. Scattering from the unextracted epidermal peel looks very similar to scattering from air, except for some differences in the low q region. Background correction of scattering data from the unextracted epidermal peel (Figure S4b) shows a q^{-4} dependence up to $q = 0.02$ Å^{-1} but no other features. This indicates that scattering for the unextracted epidermal peel is mostly from large-scale structures, such as the sample roughness.

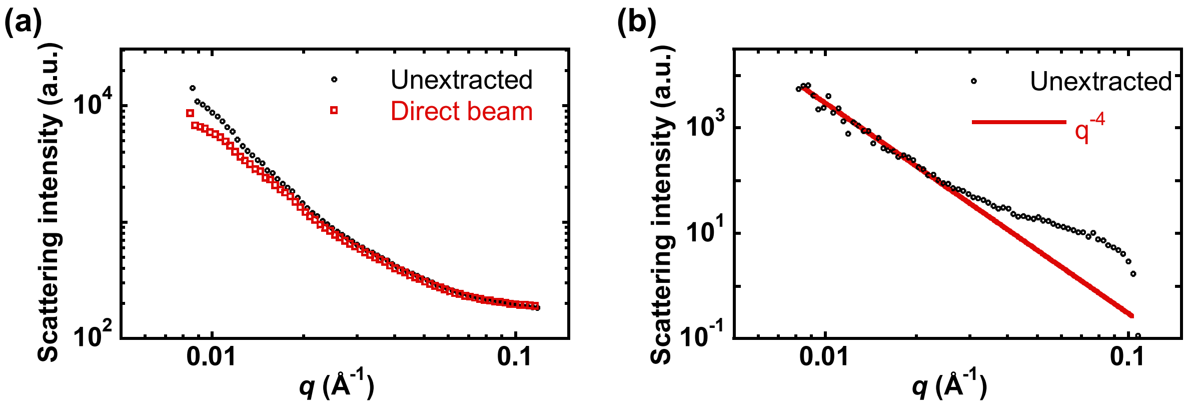

Figure S4. (a) SAXS scattering profiles of onion 11th scale epidermis (unextracted) and of air (direct beam). (b) Scattering profile of onion 11th scale epidermis after background correction.

4. Effect of sample thickness on scattering intensity

Scattering intensity (I) is a function of sample thickness (t) and sample transmittance (T):

$$I(\lambda) \propto tT(\lambda) = t\, e^{-\frac{t}{l(\lambda)}} \tag{3}$$

Sample transmittance is related to attenuation length by $T = e^{-\frac{t}{l}}$ where l is the attenuation length that depends on the wavelength λ. Attenuation is a function of energy and it increases as energy increases. Assuming the cell wall has a density of 1.5 g/cm^3, the attenuation length at 280 eV (near the carbon K-edge) is 1.75 μm (Figure S5a). The scattering intensity is maximized when the sample thickness equals the attenuation length, which can be seen from taking the derivative of Equation 3 giving $I'(t) \propto (1 - \frac{t}{l})e^{-\frac{t}{l}}$. Figure S5b shows the scattering intensity as a function of sample thickness for 10 keV X-rays. Figure S5b suggests the scattering from an epidermal peel with a thickness of 1.1 μm is much lower than the theoretical maximum scattering intensity that can be obtained for samples with larger thicknesses. Thus, we predict weak scattering from the onion epidermal cell wall at 10 keV that is potentially similar to background scattering, making it difficult to study these samples using SAXS.

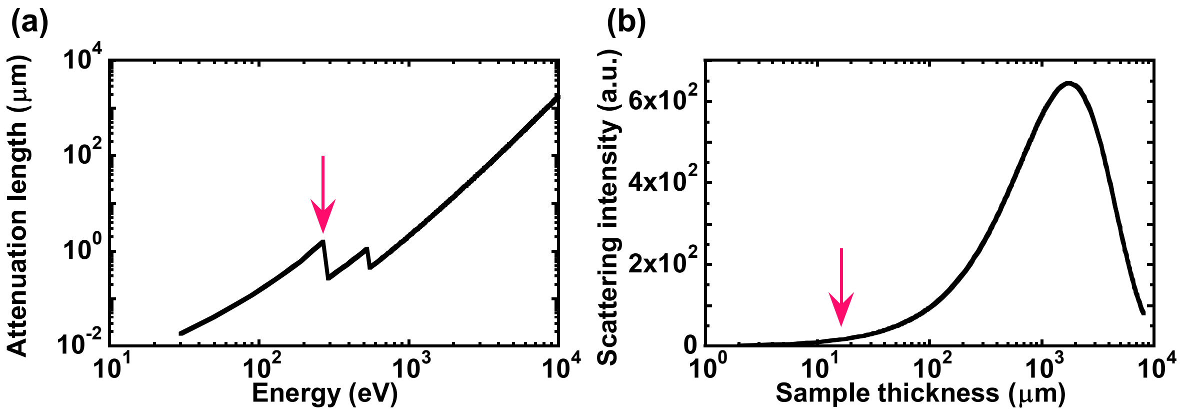

Figure S5. (a) Attenuation length of cell wall as a function of energy. The arrow corresponds to 280 eV (carbon K-edge). (b) SAXS intensity (at 10 keV) as a function of sample thickness. The arrow indicates the thickness of the onion epidermis.

5. NEXAFS and scattering contrast

We find that the calcium signal from commercially available pectin obtained from citrus (Sigma-P9436 with a 60% esterification level) is suppressed. Thus, to obtain the NEXAFS spectra for calcium-treated pectin, we used the carbon edge NEXAFS of pectin from citrus and the calcium edge NEXAFS of calcium-treated epidermal cell walls. The two datasets were merged at 320 eV (Figure S6a). Because the cell wall is only about 40% pectin (by mass),[2] we remove the expected contributions of the other 60% of polysaccharides (*e.g.*, cellulose and hemicellulose) to obtain a spectra representative of Ca-infused pectin. First, we assume that cellulose and hemicellulose do not complex with Ca ions, and therefore represent the NEXAFS spectra near the calcium L-edge (340-355 eV) as a line that connects the pre-edge (340 eV) and post-edge (355 eV), as shown in Figure S6b as a dashed black line. After subtracting the non-pectin NEXAFS (Figure S6b, dashed black line) from the calcium-treated cell wall (Figure S6b, solid blue line), the subtracted spectra were further divided by the mass fraction of pectin (0.4). The final spectra that we take as representative of calcium-treated pectin is shown in Figure S6b as a dashed red line.

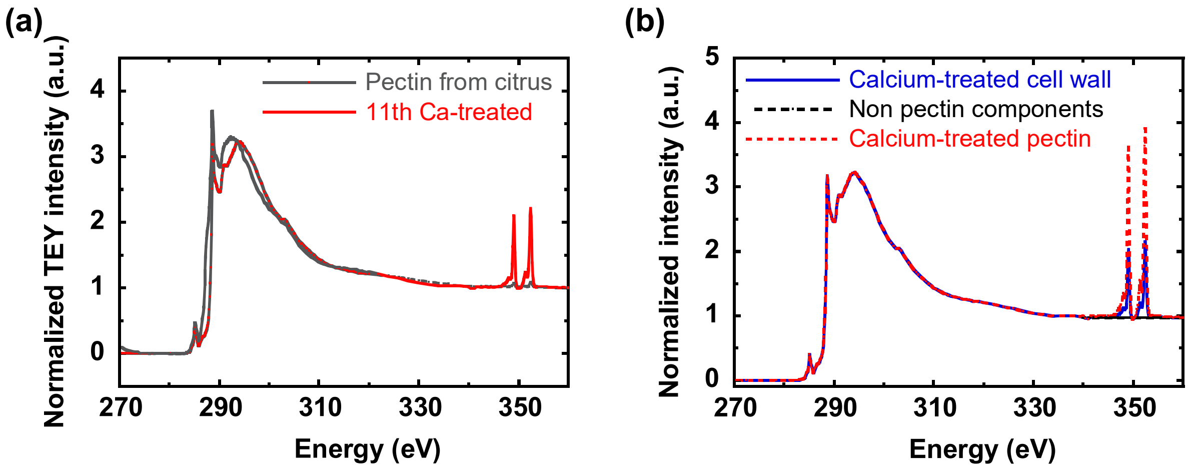

Figure S6. NEXAFS spectra of calcium treated-pectin. (a) NEXAFS spectra of pectin from citrus merged with spectra from calcium-treated 11th scale epidermis. The solid line indicates the regions of each spectra used for the merged NEXAFS spectra. (b) Calculation of calcium-treated pectin NEXAFS spectra from spectra obtained from calcium-treated cell walls.

The scattering contrast can be related to the absorptive and dispersive components of the refractive index. The absorptive component β was determined from the NEXAFS spectra shown in Figure 4a of the main text using KKcalc. For these calculations, a density of 1.599 g/cm^3 was used for cellulose[3] and a density of 1.543 g/cm^3 was used for calcium-treated pectin[4]. The β spectra is merged and extend to the entire energy range from 10 eV to 10,000 eV. The dispersive component δ is calculated using the Kramer-Kronig theorem based on β. The calculated scattering contrast between cellulose and calcium-containing pectin is shown in Figure 4b of the main text and indicates that the scattering contrast near the calcium L-edge is two orders of magnitude higher than in the hard X-ray regime.

6. 2D RSoXS Images

RSoXS 2D images for unextracted, calcium-treated, and pectate lyase-treated onion 11th scale epidermis are shown in Figure S7. The scattering anisotropy (ellipsoidal shape) of the 2D RSoXS data is due to fiber alignment of cellulose microfibrils within the epidermal cell wall and has been previously observed in SAXS studies of hypocotyl of *Arabidopsis thaliana*[5]. Anisotropy has previously been observed for other semi-crystalline filament materials as well[6].

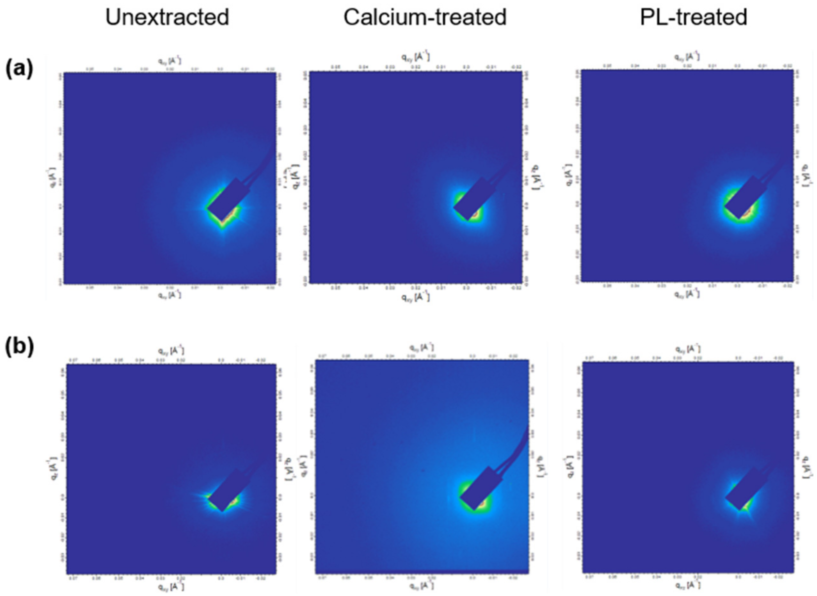

Figure S7. RSoXS 2D images of unextracted, calcium-treated, and pectate lyase (PL)-treated samples at (a) 285 eV near the carbon K-edge and (b) 352.6 eV near the calcium L-edge. Images taken in the transmission geometry.

7. Driselase digested calcium-treated epidermis

We performed RSoXS measurements near the carbon K-edge and the calcium L-edge on calcium-treated epidermis that had been digested with Driselase (Figure S8). Near the carbon K-edge, a feature is observed around q = 0.03 $Å^{-1}$. Near the calcium L-edge, the on-resonance scattering profiles (349.3 eV and 352.6 eV) are the same as the scattering profiles collected at energies which are off-resonance (345 eV and 355 eV). Because Driselase digests cell wall components and leaves the cuticle intact, this data suggests that the scattering feature near the calcium L-edge around q = 0.03 $Å^{-1}$ observed in the calcium-treated epidermis (Figure 5b of the main text) is likely not from the cuticle.

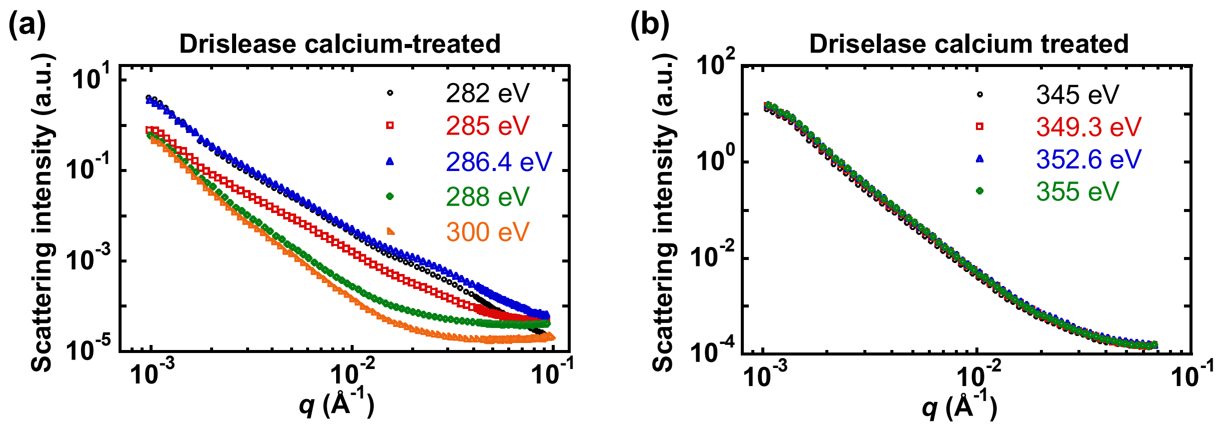

Figure S8. RSoXS profiles near (a) the carbon K-edge and (b) the calcium L-edge of calcium-treated onion 11th scale epidermis digested with Driselase. Data acquired in the transmission geometry (X-rays normal to the epidermis).

8. Total scattering intensity and scattering contrast

Total scattering intensity from RSoXS data was calculated from scattering profiles using $TSI = \int I(q)q^2dq$. For each sample, TSI at different energies was normalized to TSI at 345 eV near the calcium L-edge and 280 eV near the carbon K-edge (Figure S9).

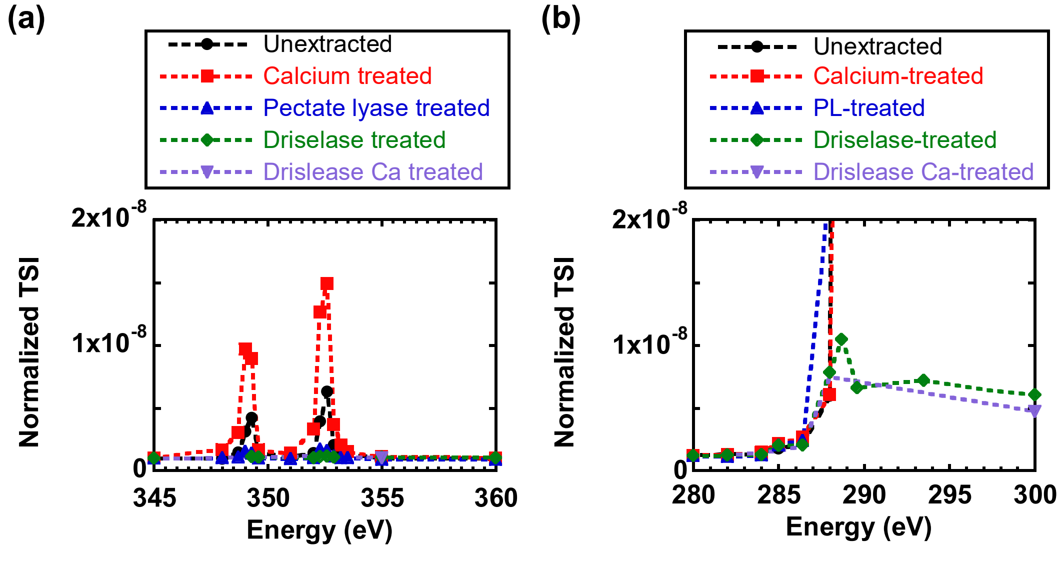

Figure S9. Normalized total scattering intensity (TSI) of onion epidermis treated with 5 different methods near (a) the calcium L-edge and (b) the carbon K-edge.

9. Comparing RSoXS data to cylindrical form factors

To explore whether the scattering feature near q = 0.03 Å^{-1} in RSoXS scattering profiles (Figure 5b of the main text) is from the form factor of cellulose microfibrils, we calculated form factors for different cylindrical shapes. Figure S10 shows that a monodisperse 36 nm diameter cylinder has a peak around q = 0.03 Å^{-1}. If that is the case, higher order reflections should appear above q = 0.03 Å^{-1}. We also examined whether the addition of polydispersity (PD) in the diameter of the cylinder can help match experimental scattering profiles. Polydispersity is defined based on the Shultz distribution $PD = \frac{d_{avg}}{\sigma^2}$, where d_{avg} is the averaged diameter and σ^2 is the variance of the distribution. By setting the polydispersity to 0.2, the feature around q = 0.03 Å^{-1} persists but there is a shoulder near q = 0.01 Å^{-1} that is not observed in RSoXS scattering profiles. When the polydispersity is increased to 0.3, no feature is apparent near q = 0.03 Å^{-1}. These data show that different cylindrical form factors cannot produce scattering profiles similar to the observed RSoXS scattering profile. Previous work has also shown that although interference between the structure factor and form factor can dampen higher order oscillations in scattering profiles, the peak width is nevertheless relatively narrow.[7, 8] Thus, we conclude that the feature around q = 0.03 Å^{-1} in RSoXS data represents the averaged interfibril spacing and not the form factor of cellulose microfibrils.

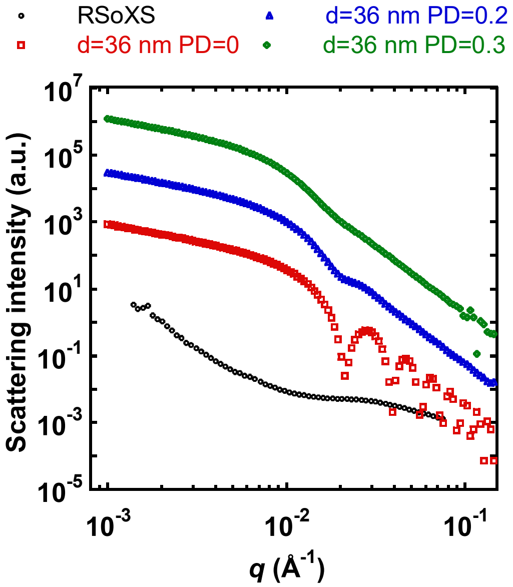

Figure S10. Comparison of RSoXS scattering profile of calcium-treated 11th scale epidermis at 349.3 eV and predicted scattering profiles for form factors with different cylindrical shapes. The cylinder form factors have a fixed diameter of 36 nm and a length of 10 μm. The polydispersity was set as 0, 0.2, and 0.3.

10. NEXAFS spectra of driselase-treated onion epidermis

To obtain the refractive index of cuticle near the C K-edge, we acquire NEXAFS data of Driselase-treated onion epidermis as shown in Figure S11.

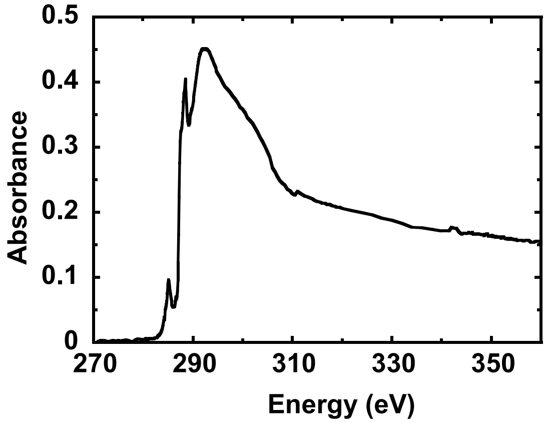

Figure S11. NEXAFS spectra of Driselase-treated epidermis. We use this spectra to calculate the optical properties of cuticle.

11. References

1. Zhang T, Zheng Y, Cosgrove DJ. Spatial organization of cellulose microfibrils and matrix polysaccharides in primary plant cell walls as imaged by multichannel atomic force microscopy. *The Plant Journal* **85**, 179-192 (2016).

2. Alexander MM, Sulebele GA. Pectic substances in onion and garlic skins. *Journal of the Science of Food and Agriculture* **24**, 611-615 (1973).

3. Sun C. True Density of Microcrystalline Cellulose. *Journal of Pharmaceutical Sciences* **94**, 2132-2134 (2005).

4. Salbu L, Bauer-Brandl A, Tho I. Direct Compression Behavior of Low- and High-Methoxylated Pectins. *AAPS PharmSciTech* **11**, 18-26 (2009).

5. Saxe F*, et al.* Measuring the distribution of cellulose microfibril angles in primary cell walls by small angle X-ray scattering. *Plant Methods* **10**, 25 (2014).

6. Masunaga H*, et al.* Multipurpose soft-material SAXS/WAXS/GISAXS beamline at SPring-8. *Polymer Journal* **43**, 471-477 (2011).

7. Kennedy CJ*, et al.* Microfibril diameter in celery collenchyma cellulose: X-ray scattering and NMR evidence. *Cellulose* **14**, 235 (2007).

8. Jakob HF, Fratzl P, Tschegg SE. Size and Arrangement of Elementary Cellulose Fibrils in Wood Cells: A Small-Angle X-Ray Scattering Study of Picea abies. *Journal of Structural Biology* **113**, 13-22 (1994).
